# Extubation failure and the use of noninvasive ventilation during the weaning process in critically ill COVID-19 patients

**DOI:** 10.5935/2965-2774.20230009-en

**Published:** 2023

**Authors:** Viviane Martins Corrêa Boniatti, Chaiane Ribeiro Pereira, Gabriela Machado Costa, Michelle Carneiro Teixeira, Alessandra Preisig Werlang, Francielle Thaisa Morais Martins, Leonardo da Silva Marques, Wagner Luís Nedel, Márcio Manozzo Boniatti

**Affiliations:** 1 Department of Critical Care, Hospital Nossa Senhora da Conceição - Porto Alegre (RS), Brazil; 2 Hospital de Clínicas de Porto Alegre - Universidade Federal do Rio Grande do Sul - Porto Alegre (RS), Brazil

**Keywords:** COVID-19, Coronavirus infections, SARS-CoV-2, Respiration, artificial, Ventilator weaning, Noninvasive ventilation, Airway extubation

## Abstract

**Objective:**

To assess the outcome of extubation in COVID-19 patients and the use of noninvasive ventilation in the weaning process.

**Methods:**

This retrospective, observational, single-center study was conducted in COVID-19 patients aged 18 years or older who were admitted to an intensive care unit between April 2020 and December 2021, placed under mechanical ventilation for more than 48 hours and progressed to weaning. Early extubation was defined as extubation without a spontaneous breathing trial and immediate use of noninvasive ventilation after extubation. In patients who underwent a spontaneous breathing trial, noninvasive ventilation could be used as prophylactic ventilatory assistance when started immediately after extubation (prophylactic noninvasive ventilation) or as rescue therapy in cases of postextubation respiratory failure (therapeutic noninvasive ventilation). The primary outcome was extubation failure during the intensive care unit stay.

**Results:**

Three hundred eighty-four extubated patients were included. Extubation failure was observed in 107 (27.9%) patients. Forty-seven (12.2%) patients received prophylactic noninvasive ventilation. In 26 (6.8%) patients, early extubation was performed with immediate use of noninvasive ventilation. Noninvasive ventilation for the management of postextubation respiratory failure was administered to 64 (16.7%) patients.

**Conclusion:**

We found that COVID-19 patients had a high rate of extubation failure. Despite the high risk of extubation failure, we observed low use of prophylactic noninvasive ventilation in these patients.

## INTRODUCTION

A large proportion of coronavirus disease 2019 (COVID-19) patients progress to a more severe form of the disease and require hospitalization in an intensive care unit (ICU).^(1,2)^ Most of these patients require mechanical ventilation (MV) and have a protracted clinical course marked by difficulty in ventilator liberation.^(3,4)^ Data on successfully weaning COVID-19 patients from MV are limited.

The decision to extubate a patient can be quite difficult. Very early extubation can increase the risk of reintubation, prolong the ICU length of stay and increase mortality.^(5,6)^ On the other hand, an unnecessary delay in extubation can also lead to complications associated with a longer MV duration and inefficient use of intensive care resources.^(7,8)^

The use of noninvasive ventilation (NIV) during weaning has been extensively investigated in non-COVID-19 patients. Early extubation followed by immediate NIV, prophylactic NIV after extubating a patient who tolerated a spontaneous breathing trial (SBT), and NIV as rescue therapy for postextubation respiratory failure are applied during weaning from MV.^(9-11)^ To date, there have been very few investigations on the role of NIV in weaning COVID-19 patients from MV.

This study aimed to assess the outcome of extubation in COVID-19 patients and the use of NIV in the weaning process.

## METHODS

This retrospective, observational, single-center study was conducted in the ICU of *Hospital Nossa Senhora da Conceição*, located in Porto Alegre, Brazil, from April 2020 to December 2021. During the COVID-19 pandemic, the hospital increased the number of ICU beds and allocated 50 ICU beds exclusively for COVID-19 patients at the peak of the pandemic. This study was approved by the Research Ethics Committee of the hospital (no. 4164341). Due to the retrospective nature of the study, the need for informed consent was waived.

Patients aged 18 years or older who were admitted to the ICU with COVID-19 confirmed by reverse transcriptase polymerase chain reaction (RT‒PCR) or antigen testing for SARS-CoV-2, placed under MV for a period of at least 48 hours, and progressed to weaning were included. Patients who were self-extubated or accidentally extubated, who underwent tracheostomy before an extubation attempt, or who died before weaning were excluded.

The criteria to start the weaning process were as follows: improvement or resolution of the patient’s condition by MV; body temperature below 38.5°C; hemoglobin ≥ 8g/dL; no or minimal doses of vasoactive drugs and sedatives; arterial oxygen pressure (PaO_2_) > 60mmHg or peripheral oxygen saturation (SpO_2_) > 90%; fraction of inspired oxygen (FiO_2_) < 0.4; and positive end-expiratory pressure (PEEP) ≤ 8cmH_2_O. Patients who tolerated pressure support ventilation (PSV) mode with a PEEP of 5 - 8cmH_2_O and pressure above PEEP of 7 - 14cmH_2_O underwent an SBT, which was performed in PSV mode with pressure over PEEP ≤ 8cmH_2_O and PEEP ≤ 5cmH_2_O or with a T-piece for 30 minutes. The criteria for SBT intolerance were agitation, anxiety, low level of consciousness (Glasgow coma scale score < 13), respiratory rate > 35/minute and/or use of accessory muscles, SpO_2_ < 90%, heart rate > 140 beats/minute or > 20% of the baseline, systolic blood pressure < 90mmHg, or the development of arrhythmia. Patients who tolerated the SBT were extubated. For patients who failed the SBT, the assisted ventilation mode was reapplied, and a new SBT was performed after 24 hours.

The primary outcome was extubation failure, which was defined as the need for reintubation during the ICU stay. Secondary outcomes were extubation failure within 48 hours and 96 hours of extubation, ICU mortality, and in-hospital mortality.

Early extubation was defined as extubation without an SBT and immediate use of NIV after extubation.^(9)^ NIV applied in this way has been proposed as an alternative to invasive MV in patients who are not yet ready to be extubated (i.e., NIV to facilitate weaning).^(9)^ These two criteria were necessary to define early extubation, i.e., not performing an SBT and using NIV immediately after extubation. These patients had a pressure over PEEP ≤ 14cmH_2_O (criterion to initiate weaning) and > 8cmH_2_O (≤ 8cmH_2_O was considered to indicate an SBT). In the patients who underwent an SBT, NIV could be used as prophylactic ventilatory assistance when started immediately after extubation (prophylactic NIV) or as rescue therapy in cases of postextubation respiratory failure (therapeutic NIV).

The clinical and demographic data collected were as follows: age; sex; Simplified Acute Physiology Score 3 (SAPS 3); use of NIV as preintubation support; preintubation PaO_2_/FiO_2_; ventilatory parameters on the first day of MV (PEEP and plateau pressure); use of NIV (prophylactic, therapeutic, or associated with early extubation) after extubation; fluid balance in the 24 hours before extubation; weaning time (time between the first SBT and extubation); duration of invasive MV; and ICU or hospital mortality.

Continuous variables are described as means and standard deviations or medians and interquartile ranges, and categorical variables are described as absolute numbers and percentages. Student’s t or Wilcoxon Mann-Whitney tests were used for continuous variables, and Fisher’s exact test was used for categorical variables. A Cox proportional hazards model was constructed to evaluate whether prophylactic NIV was associated with extubation failure. Prophylactic NIV was maintained as a variable of interest in the model. Other variables defined post hoc were those plausibly associated with the primary outcome (age, fluid balance, SAPS 3, and duration of MV). The assumption of linearity of independent variables with log-odds was assessed by Box-Tidwell transformation. A logarithmic transformation was performed on nonlinear independent variables. The transformed variables were then included in the Cox proportional hazards model as independent variables. For all comparisons, a p value < 0.05 was considered statistically significant. Statistical analyses were performed using IBM Statistical Package for the Social Sciences (SPSS), version 20.0 (IBM Corp., Armonk, NY, USA) and R 3.6.2 (The R Foundation).

## RESULTS

**Figure 1:**
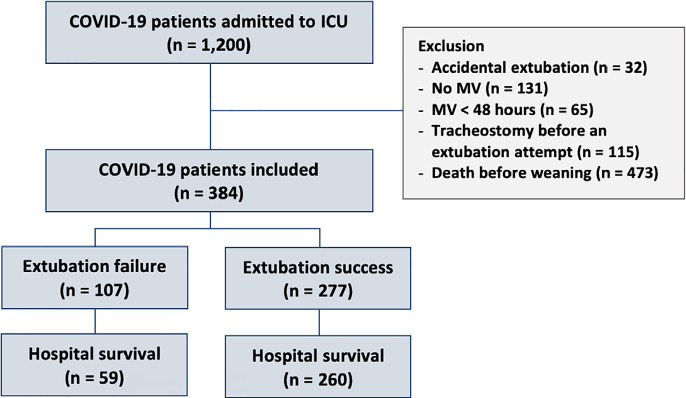
Flowchart for inclusion of patients in the study.

During the study period, 1200 patients with a confirmed diagnosis of COVID-19 were admitted to the ICU. Of these, 816 were excluded from the study. Thus, a total of 384 patients were included in the final analysis (Figure 1).

MV - mechanical ventilation; ICU - intensive care unit.

Demographic characteristics and clinical parameters are described in table 1. One hundred thirty-seven (35.7%) patients received noninvasive support (prophylactic, therapeutic, or associated with early extubation) after extubation. Extubation failure was observed in 107 (27.9%) patients. The failure rate was 19.5% and 24.0% at 48 and 96 hours after extubation, respectively. The ICU mortality rate was 39.4% in patients who required reintubation during their ICU stay. Intensive care unit mortality did not differ between patients requiring reintubation within less than 48 hours (34.2%) or after more than 48 hours (47.2%) (p = 0.214).

**Table 1:** Characteristics of mechanically ventilated COVID-19 patients

	Patients who received noninvasive support after extubation (n = 137)	Patients who did not receive noninvasive support after extubation (n = 247)	p value
Age (years)	53.8 ± 13.1	53.9 ± 14.9	0.988
Sex, male	79 (57.7)	133 (53.8)	0.471
SAPS 3	64.3 ± 13.2	61.2 ± 15.6	0.091
PEEP in the first day (cmH_2_O)	12.4 ± 3.4	11.2 ± 3.0	< 0.001
Plateau pressure in the first day (cmH_2_O)	27.0 (24.0 - 30.0)	26.0 (24.0 - 29.0)	0.062
PaO_2_/FiO_2_ preintubation	84.0 (69.0 - 118.0)	115.5 (82.0 - 209.0)	< 0.001
NIV preintubation	58 (42.3)	60 (24.3)	< 0.001
Fluid balance - last 24 hours (mL)	-4.0 (-544.0 - 900.0)	0.0 (-666.3 - 499.3)	0.412
Duration of weaning (days)	0.0 (0.0 - 0.0)	0.0 (0.0 - 1.0)	0.261
Duration of MV (days)	12.6 ± 7.0	10.0 ± 6.4	< 0.001
Reintubation at 48 hours	34 (24.8)	41 (16.6)	0.052
Reintubation at 96 hours	44 (32.1)	48 (19.4)	0.005
Reintubation in ICU	56 (40.9)	51 (20.6)	< 0.001
ICU mortality	23 (16.8)	28 (11.3)	0.132
Hospital mortality	30 (21.9)	35 (14.2)	0.053

SAPS 3 - Simplified Acute Physiology Score 3; PEEP - positive end-expiratory pressure; PaO_2_ - partial pressure of oxygen; FiO_2_ - fraction of inspired oxygen; NIV - noninvasive ventilation; MV - mechanical ventilation; ICU - intensive care unit. The results are expressed as the mean ± standard deviation, n (%) or median (interquartile range).

**Figure 2:**
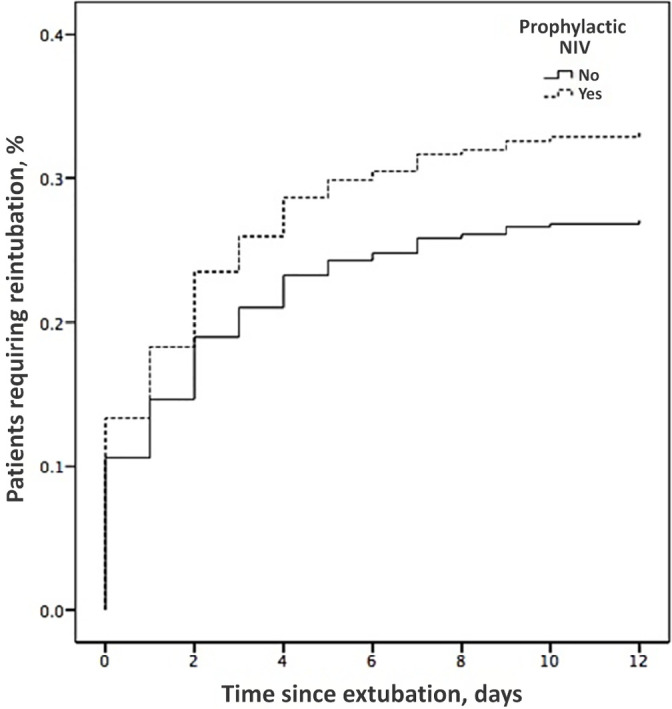
Kaplan-Meier analysis of the time from extubation to reintubation, including all patients and dividing according to prophylactic noninvasive ventilation use.

Regarding the use of NIV, 47 (12.2%) patients received prophylactic NIV. Of these, 16 (34.0%) experienced extubation failure. The extubation failure rate in the 337 patients who did not receive prophylactic NIV was 27.0% (n = 91; p = 0.304) (Figure 2). The Cox regression analysis showed that prophylactic NIV was not associated with extubation failure after multivariable adjustment (hazard ratio - HR 1.27; 95%CI 0.75 - 2.17; adjustment hazard ratio - aHR 0.57; 95%CI 0.16 - 1.98). In 26 (6.8%) patients, early extubation was performed with the immediate use of NIV. There was no difference in the mean duration of MV between the patients who underwent early extubation (11.6 ± 6.1 days) and patients who underwent an SBT (10.9 ± 6.8 days) (p = 0.586). Noninvasive ventilation for the management of postextubation respiratory failure was provided to 64 (16.7%) patients; 32 (50.0%) required reintubation.

NIV - noninvasive ventilation.

## DISCUSSION

We observed an extubation failure rate of 27.9% in patients with COVID-19. Interestingly, despite the high risk of extubation failure, we observed low use of prophylactic NIV in these patients.

The extubation failure rate found in our study was similar to the previously described rate described in COVID-19 patients when considering reintubation during the ICU stay as a criterion (22.1% to 33.1%).^(12-14)^ Guzatti et al. found that the extubation failure rate was approximately three times higher during the ICU stay (22.1%) than during the 48-hour period (7.8%) after extubation and hypothesized that COVID-19 patients experience late extubation failure.^(13)^ In our study, we did not find this trend of late extubation failure because 81% of the patients who required reintubation were reintubated within the first three days after extubation.

A longer MV duration is a characteristic that differs from non-COVID-19 patients and may be associated with a higher rate of extubation failure. The mean duration of MV in this study was 10.9 ± 6.7 days, which is similar to that in other studies of COVID-19 patients^(12-14)^ and is more than double that in non-COVID-19 patients.^(15)^

Little is known about the use of NIV in weaning COVID-19 patients. The strategy of early extubation with immediate NIV was associated with a reduced invasive MV duration in a meta-analysis of studies of non-COVID-19 patients.^(9)^ Thille et al. found a reduction in the extubation failure rate with prophylactic NIV use in non-COVID-19 patients at high risk of extubation failure,^(10)^ and a recent network meta-analysis confirmed this finding.^(16)^ Finally, NIV to treat postextubation respiratory failure in non-COVID-19 patients also reduced the need for reintubation in a recent randomized clinical trial.^(11)^ There are limited data on these three modes of NIV use during weaning in COVID-19 patients. In an observational study, Cammarota et al. found that the strategy of early extubation followed by immediate NIV was chosen in 54.5% of patients and reduced both the duration of invasive MV and the need for reintubation.^(12)^ In this study, prophylactic NIV was used in 60% of patients undergoing standard weaning, and 29% of these patients received NIV as a salvage treatment for postextubation respiratory failure. In our study, the strategy of early extubation followed by immediate NIV was chosen infrequently and was not associated with a shorter invasive MV duration. Furthermore, the rates of prophylactic NIV (12.2%) and NIV as rescue therapy for postextubation respiratory failure (16.7%) were much lower in our study. The low frequency of patients supported with NIV after extubation is probably related to the uncertainty of the benefits of this strategy in this group of patients. Only one study^(12)^ evaluated the use of early extubation followed by immediate NIV, and no studies have evaluated the use of prophylactic NIV after extubation. It remains unclear whether prophylactic NIV after extubation should be used more frequently in COVID-19 patients. However, the rationale for increasing its use is based not only on the fact that these patients could be considered at high risk for extubation failure but also on the evidence of benefits from this strategy in high-risk non-COVID-19 patients.

This study has some limitations. First, the observational and retrospective design does not allow for the establishment of a cause‒effect association between the various factors evaluated and the outcome of extubation. Second, it is a single-center study with a small number of patients, which limits the generalizability of the results. Third, data for some variables, such as ICU-acquired weakness and need for aspiration, could not be collected. Finally, the experience gained over time and the availability of resources may have influenced the use of NIV.

## CONCLUSION

We found that COVID-19 patients had a high rate of extubation failure. This highlights the need for careful monitoring of these patients after extubation and the importance of identifying and addressing risk factors for extubation failure in this population. Interestingly, despite the high risk of extubation failure, we observed low use of prophylactic noninvasive ventilation in these patients. Future studies are needed to investigate the reasons behind this underutilization and to determine whether prophylactic noninvasive ventilation can reduce the risk of extubation failure in patients with COVID-19.
